# The Global Emerging Infection Surveillance and Response System (GEIS), a U.S. government tool for improved global biosurveillance: a review of 2009

**DOI:** 10.1186/1471-2458-11-S2-S2

**Published:** 2011-03-04

**Authors:** Kevin L Russell, Jennifer Rubenstein, Ronald L Burke, Kelly G Vest, Matthew C Johns, Jose L Sanchez, William Meyer, Mark M Fukuda, David L Blazes

**Affiliations:** 1 Armed Forces Health Surveillance Center, 11800 Tech Rd, Silver Spring, MD 20904, USA

## Abstract

The Armed Forces Health Surveillance Center, Global Emerging Infections Surveillance and Response System (AFHSC-GEIS) has the mission of performing surveillance for emerging infectious diseases that could affect the United States (U.S.) military. This mission is accomplished by orchestrating a global portfolio of surveillance projects, capacity-building efforts, outbreak investigations and training exercises. In 2009, this portfolio involved 39 funded partners, impacting 92 countries. This article discusses the current biosurveillance landscape, programmatic details of organization and implementation, and key contributions to force health protection and global public health in 2009.

## Introduction and background

Despite optimism in the 1960s that mankind had conquered infectious diseases, the world has repeatedly confronted the reality of its continued vulnerability. Two landmark Institute of Medicine (IOM) reports outlined these vulnerabilities [1,2]. Recent events emphasize the wisdom of these documents, and the fact that the global community must unite to address emerging infectious diseases.

**Figure 1:**
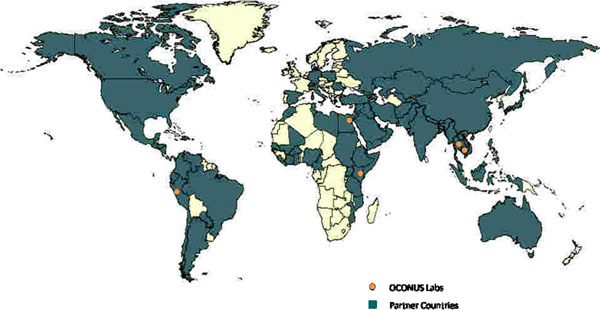
Global reach of AFHSC-GEIS partnership through surveillance, capacity building or training initiatives

The first of two IOM reports, released in 1992, highlighted the potential role of Department of Defense (DoD) overseas laboratories in addressing the vulnerabilities of emerging infections. DoD has a long history of medical research and development, much of which has been performed through a network of overseas laboratories. Although their geographic locations have changed through time, five laboratories were in operation in 2009: Cairo, Egypt; Nairobi, Kenya; Bangkok, Thailand; Lima, Peru; and Jakarta, Indonesia in 2009 (Figure 1) [3]. Historically, the role of these laboratories was limited almost exclusively to the research and development of products, such as vaccines, antimicrobials or diagnostics, that would benefit the health of DoD forces throughout the world. Surveillance for infectious diseases, however, was minimal. Between 1992 and 1996, numerous documents and communications within DoD recognized the need for global emerging infection surveillance initiatives leveraging these overseas laboratories, and emphasized the commitment of DoD to these endeavors.

In 1996, the Executive Office of the President of the United States issued a Presidential Decision Directive (NSTC-7) stating that current capabilities were inadequate to protect the U.S. or global public health communities from emerging infectious disease (EID) threats [4]. DoD was again specifically noted among various federal agencies as having global presence and expertise that could be leveraged to help improve worldwide EID surveillance and preparedness. With these events, the DoD Global Emerging Infections Surveillance and Response System (DoD-GEIS) was established, thereby expanding the mission of DoD to address threats posed to the U.S. and other nations by newly emerging and re-emerging infectious diseases. This was a timely development: The next decade brought SARS, West Nile virus and avian influenza, to name a few, and more recently, the H1N1 influenza virus emerged in 2009 as a pandemic threat.

In 2008, DoD-GEIS became a Division of the Armed Forces Health Surveillance Center (AFHSC) by direction of the deputy secretary of defense [5]. This move centralized DoD-wide healthcare surveillance initiatives with domestic and overseas laboratory surveillance efforts. In 2009, AFHSC-GEIS provided direction, funding and oversight to a network of 39 partners (Table 1) at approximately 500 sites. Ninety-two countries were impacted with either active surveillance, capacity-building initiatives or participation in training exercises (Figure 1). This paper will summarize implementation of this global DoD laboratory surveillance network and its contributions in 2009, and discuss potential for the future as the U.S. government becomes increasingly proactive in global biosurveillance.

**Table 1:** Global partners 2009 and region of engagement

	FY09 Funded Partners	Primary Countries Engaged
1	**65^th^ Medical Brigade – Korea**	Republic of South Korea
2	**Armed Forces Institute of Pathology – Washington, DC**	Global U.S. DoD visibility
3	**Armed Forces Research Institute of Medical Sciences – Bangkok, Thailand**	Thailand, Cambodia, Lao PDR, Philippines, Nepal & Bhutan and US Embassies and Consulate offices throughout Southeast Asia
4	**Australian Army Malaria Institute – Enoggera, Australia**	Australia, Vanuatu & Solomon Islands
5	**Center for Disaster and Humanitarian Assistance Medicine – Bethesda, MD**	Numerous with global distribution
6	**DoD Veterinary Food Analysis & Diagnostic Laboratory – Fort Sam Houston, TX**	Overseas food & water production facilities with DoD procurement contracts and US military installations supporting Military Working Dogs and food facilities
7	**University of Iowa – Iowa City, IA**	Thailand, Cambodia, Mongolia, Nigeria & Romania
8	**Johns Hopkins University Applied Physics Laboratory – Laurel, MD**	US military installations; Philippines, Peru & Cambodia
9	**Landstuhl Regional Medical Center – Germany**	US military treatment facilities in Southwest Asia, Germany, Italy, Belgium, Spain, United Kingdom, Turkey, Poland & Ukraine
10	**National Aeronautics and Space Administration – Greenbelt, MD**	Numerous with distribution primarily in Africa, Southeastern Europe and Central Asia
11	**Naval Health Research Center – San Diego, CA**	US military training facilities; 2^nd^, 3^rd^ and 7^th^ US Naval Fleets and deployed US Naval & Marine Corps personnel in Western Pacific region; US/Mexico border clinics with US CDC
12	**Navy and Marine Corps Public Health Center – Portsmouth, VA**	US military treatment facilities within the military health system (MHS)
13	**Navy Environmental Preventive Medicine Unit – 2 – Norfolk, VA**	US military treatment facilities in Djibouti, Kuwait, Qatar, Bahrain, Iraq & Afghanistan; deployed US Naval & Marine Corps personnel in Southwest Asia & shipboard activities in the Atlantic
14	**Navy Medical Research Center – Silver Spring, MD**	Numerous with global distribution
15	**Navy Medical Research Center Detachment – Lima, Peru**	Eleven countries in Central & South America
16	**Navy Medical Research Unit – 3 – Cairo, Egypt**	Thirty-four countries in West/North Africa, the Middle East & Central Asia and deployed US Forces throughout Southwest Asia and Eastern Europe
17	**Navy Medical Research Unit-2 – Jakarta, Indonesia**	Cambodia, Lao PDR, Indonesia & Singapore
18	**Pacific Air Force – Hickman AFB, HI**	Lao PDR & Vietnam
19	**Public Health Command Region - Europe (formerly CHPPM-Eur) – Landstuhl, Germany**	US military treatment facilities in Southwest Asia, Germany, Italy, Belgium, Spain, United Kingdom, Turkey, Poland & Ukraine
20	**Public Health Command Region - Pacific (formerly CHPPM-Pac) – Camp Zama, Japan**	US military treatment facilities & deployed US Forces in Japan & South Korea
21	**Public Health Command Region - South (formerly CHPPM-South) – Fort Sam Houston, TX**	US military treatment facilities; civilian MoH laboratory centers in Guatemala, El Salvador, Honduras, Nicaragua & Panama
22	**San Antonio Military Medical Center (formerly BAMC) – San Antonio, TX**	US military treatment facilities in Southwestern US
23	**U.S. Army Medical Research Institute of Infectious Disease – Fort Detrick, MD**	US military treatment facilities & overseas VHF laboratory in Sierra Leone
24	**U.S. Army Medical Research Unit – Kenya – Nairobi, Kenya**	Kenya, Tanzania, Uganda, Cameroon & Nigeria
25	**U.S. Northern Command – Colorado Springs, CO**	US military installations & coordination with Mexico and Canadian counterparts
26	**U.S. Southern Command – Miami, FL**	Deployed US Forces throughout Latin America
27	**UCLA/Global Viral Forecasting Initiative – San Francisco, CA**	Cameroon
28	**Uniformed Services University of the Health Sciences – Bethesda, MD**	US military treatment facilities & overseas military research laboratories in Peru, Egypt, Kenya, Thailand, Indonesia & Korea
29	**United States Africa Command – Stuttgart, Germany**	Deployed US Forces throughout Africa
30	**United States Air Force School of Aerospace Medicine – Wright Patterson AFB, Ohio**	US Military MTF sentinel sites around the world
31	**United States Central Command – MacDill AFB, FL**	Deployed US Forces throughout Southwest and Central Asia
32	**United States European Command –Stuttgart, Germany**	Deployed US Forces throughout Europe & Central Asia
33	**United States Pacific Command – Camp H.M. Smith, HI**	Deployed US Forces throughout Far East, Southeast Asia & the Pacific
34	**Walter Reed Army Institute of Research, Division of Bacterial Diseases – Silver Spring, MD**	US military treatment facilities & overseas military research laboratories in Peru, Egypt, Kenya, Thailand & Indonesia
35	**Walter Reed Army Institute of Research, Division of Clinical Trials – Silver Spring, MD**	Support to global system
36	**Walter Reed Army Institute of Research, Division of Entomology – Silver Spring, MD**	Numerous with global distribution
37	**Walter Reed Army Institute of Research, Division of Experimental Therapeutics – Silver Spring, MD**	Support to global system
38	**Walter Reed Army Institute of Research, Division of Virus Diseases – Silver Spring, MD**	Over 35 US embassies & deployed military personnel worldwide; overseas military research laboratories in Peru & Thailand
39	**Walter Reed Army Medical Center - Washington, DC**	Support to military personnel deployed to Iraq & Afghanistan

## The current global biosurveillance landscape

In addition to AFHSC-GEIS, many other DoD, U.S. government and U.S. nongovernmental organizations engage in surveillance or capacity-building activities throughout the world [6,7]. In 2009, the U.S. Agency for International Development (USAID) spent more than $1.7 billion on health and over $1.4 billion on humanitarian assistance [8]. Fiscal year 2009 appropriations by the U.S. Congress totaled $33.7 million for the Centers for Disease Control and Prevention’s (CDC) Global Disease Detection Program, the principal and most visible CDC program for developing and strengthening global public health capacity to rapidly identify and contain disease threats from around the world. The total budget for CDC’s global health programs in fiscal year 2009—including the Global AIDS Program, Global Immunization Program, Global Malaria Program and others—was $308.8 million [9]. The U.S. Department of State’s Biological Engagement Program (BEP) received congressional appropriations of $27 million in fiscal year 2009 to engage scientists internationally on issues related to disease surveillance and detection, biosafety and biosecurity. The U.S. Department of Agriculture (USDA) addresses animal health surveillance in the U.S., but is also engaged internationally in capacity building, research and biological control, and outbreak response, with a focus on identifying and evaluating biological agents that could impact global commerce of agricultural products [10]. USDA is also the official U.S. representative to the World Organisation for Animal Health (OIE).

Through Defense Health Program funding, the assistant secretary of defense for health affairs provides $52 million annually to AFHSC-GEIS. The assistant to the secretary of defense for nuclear and chemical and biological defense programs recently embraced emerging infections as a threat to national security, placing global surveillance also within the scope of that organization [11]. Implemented largely through the Defense Threat Reduction Agency, historically that organization’s focus has been *threat-agent* reduction and containment in the former Soviet Union. Authorization to extend globally and beyond threat agents is in process and will be conducted in part through the agency’s Cooperative Biological Engagement Program. This is likely to result in an additional infusion of resources into DoD’s global surveillance efforts. Although not directly involved in surveillance efforts, the Military Infectious Disease Research Program (MIDRP) has a mission of protecting the U.S. military against infectious diseases through research and development projects designed to develop products for mitigation, such as vaccines, medications or vector-control systems. Excluding pediatric vaccines, DoD had a major role in developing and licensing 40 percent of currently available vaccines for adults in the U.S. [12]. Most drugs licensed for the treatment of malaria were also products of DoD research and development [13,14]. AFHSC-GEIS surveillance provides baseline infectious disease risk data that directly influences priorities and viable geographic locations for the conduct of various projects within the MIDRP.

Much of the justification for engagement by the U.S. government in this work falls under the category of “health diplomacy.” The meaning of “global health diplomacy” can be controversial, but a commonly accepted definition by the University of California at San Francisco is “political change activity that meets the dual goals of improving global health and maintaining and improving international relations abroad, particularly in conflict areas and resource-poor environments.”

The involvement of DoD partners throughout the world in implementing this program can clearly be seen as serving a global health diplomacy role. By conducting surveillance and capacity building and assisting with training and outbreak investigations, all integrated into the functions and capabilities of host-country agencies, relationships are forged and trust is developed. International relations abroad are improved. Other DoD organizations work in this broad field of health diplomacy, but less directly in active biosurveillance.

Funding avenues and oversight for these different U.S. government health and surveillance initiatives are independent of each other, and coordination is complex. In a recent publication, the Center for Strategic and International Studies commented that with expanding efforts, agencies should leverage the existing successful programs, and seek a “unity of effort.” [15]. The release in November 2009 of the National Strategy for Countering Biological Threats (Presidential Policy Directive-2) also emphasizes the need for coordination: “No single stakeholder can fully address the challenge of biological threats on its own” [16]. This document uses similar terminology as many of the mid-1990s documents that resulted in the development of DoD-GEIS.

## Why the Department of Defense?

The global laboratory assets of DoD have long been recognized as valuable platforms from which to conduct biosurveillance. Each laboratory is “sponsored” in-country by either the Ministry of Defense or Ministry of Health. In addition, close working relationships exist with other components of the host and neighboring countries’ governments and academic institutions. Leveraging and empowering these relationships is a formula for success with expanded activities. Maintaining personnel at these military laboratories has also proven sustainable over time, when other U.S. government programs found this to be difficult. DoD’s unique ability to provide valuable logistical support is a factor, as is its global integrated health care system meeting the health needs of uniformed families throughout the world that can help determine exposures and risk. The synergy between this system and the DoD laboratory system is becoming clear now that both organizations exist at AFHSC.

Another reason for DoD engagement in these endeavors lies in DoD’s mission to “deter war and protect the security of our country” [17]. Combat aggressors are but one threat to our security. In the words of James Baldwin, novelist and civil rights activist, “The most dangerous creation of any society is the man who has nothing to lose.” Endemic diseases in many resource-poor settings are a cause of instability. Each year, more than 1.6 million people die from diarrheal disease, 800,000 from malaria and 20,000 from dengue fever [18-20]. This burden of known endemic diseases imposes an economic toll and resulting instability. In contrast, emerging infections, whether naturally occurring or the result of human introductions, can result in social unrest and instability on a scale quite out of proportion to the level of risk they introduce [21]. Though new agents have the potential for high morbidity and mortality, fear can have an even greater impact.

One example is the severe acute respiratory syndrome (SARS) that rapidly spread around the world in 2003. By midyear, 8,098 individuals were known to have been infected with SARS, resulting in 774 deaths. In the scope of international infectious diseases, this toll on human life was minor. However, the economic impact is estimated at between $40 billion and $52 billion [22]. Likewise, 17 infections and five deaths were attributed to the intentional anthrax attacks in 2001. These small numbers do not adequately speak to the crippling disruption of services or huge economic losses incurred. According to a recent IOM report, “Global health and national security are inexorably intertwined” [23].

Considering these facts, the enormous importance of early identification and mitigation of infectious disease threats is a critical component of a national defense strategy to “deter war and protect the security of our country.”

## Implementation of the AFHSC/GEIS program: methods

**Figure 2:**
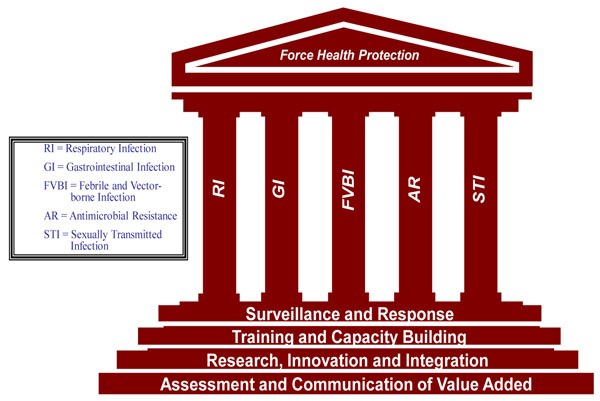
Priority pillars and strategic goals of the AFHSC-GEIS program

The GEIS system functions on a model of “priority pillars” and “strategic steps” (Figure 2). The priority infectious disease pillars include respiratory, gastrointestinal, febrile and vector-borne, antimicrobial-resistant, and sexually transmitted infections. The strategic steps include surveillance and response; training and capacity building; research, innovation and capacity building; and communication of value added. Through integrated implementation of the strategic steps, a comprehensive yet flexible program is created which recognizes the needs of host and partner countries.

Funding for global surveillance initiatives in 2009 was approximately $52 million; $40 million of this was for pandemic/avian influenza initiatives (respiratory pillar), with the remainder available for surveillance in the other EID pillars. In preparation for distribution of these funds, a request for proposals was circulated among partner laboratories in the third quarter of fiscal 2008. A total of 198 proposals were received and evaluated by an internal review board of AFHSC staff. Each proposal was evaluated based on a) potential to fill a critical gap in public health programs, b) likelihood of tri-service or DoD-wide benefits, c) facilitation of timely public health actions, d) responsiveness to critical operational theater or regional needs, e) quality of epidemiology and science, f) leveraging of existing strengths, and g) accessibility of nonfiscal resources needed for execution. In addition, prior performance of the requesting organization and principal investigator was taken into consideration. Proposals were ranked based on scores received, and a cutoff level for funding was assigned based on score and available funding. An external review board, not associated with AFHSC-GEIS and representing all three major uniformed services, reviewed overall funding decisions and provided recommendations. Finally, GEIS and AFHSC directors were briefed and given the opportunity for input. Of the 198 proposals received, full or partial funding was available for the top-ranked 66 percent (130 of 198 proposals), and 56 percent of requested funding was allocated.

## Communication of value added

Communication within and outside the network was conducted in a variety of ways: required quarterly reports, monthly conference calls with awarded partners, consolidated DoD influenza reports (with variable frequency from daily to weekly during the emerging 2009 H1N1 pandemic), site visits with program reviews, peer-reviewed publications, and presentations at multiple DoD and civilian international conferences. Results were reported only with local-host or partner-country notification and concurrence. In general, the information requested and shared by the GEIS network was aggregate in nature. GEIS does not archive extensive data sets from partners or host countries. Analysis and interpretation is largely done by the partner conducting the work, in collaboration with the host country, and with ultimate consideration of national sovereignty and transparency in the process.

The central coordination of this global DoD surveillance system afforded multiple opportunities for enhanced utilization of partner capabilities, as well as concise information sharing with other DoD organizations and external agencies (Table 2). The many examples share a central theme of leveraging global visibility and connecting needs with capabilities.

**Table 2:** Specific examples of central coordination, fiscal year 2009

**1. Funding NMRC for development, production and sharing within partner network of rickettsial diagnostic tests**
**2. Funding USAMRIID for development, production and sharing within partner network of lassa fever and other select agent diagnostic tests**
**3. Funding BAMC for development, production and sharing within partner network of leptospiral diagnostic tests**
**4. Facilitation of sample sharing for advanced characterizations**
**a. Partner H1N1 samples to WRAIR for full-genome sequencing**
**b. Shipboard outbreak respiratory and serum samples to NHRC for determination of etiology and immune status**
**5. Facilitation of ongoing discussions and updates on outbreaks among host-country populations and U.S. military beneficiaries in all regions under surveillance**
**6. Facilitation of brief summaries and updates of activity related to the 2009 pandemic of A/H1N1**
**a. Provided a forum for case reporting and regional surveillance findings among network labs and near partners within the countries (InstitutPasteur, PAHO, academic partners)**

NMRC: Naval Medical Research Center; USAMRIID: U.S. Army Medical Research Institute of Infectious Diseases; BAMC: Brooke Army Medical Center; WRAIR: Walter Reed Army Institute of Research; PAHO: Pan American Health Organization

Communication with the World Health Organization (WHO) and CDC is a priority, with a DoD liaison positioned in both organizations to facilitate bilateral information exchange. The value added to these two organizations by the GEIS network is clear in the examples of the WHO reference laboratory status of Naval Medical Research Unit Number 3 (NAMRU-3) in Cairo, Egypt, and U.S. Army Medical Research Unit-Kenya (USAMRU-K). Both laboratories were highly leveraged in training and laboratory capacity building during the 2009 H1N1 pandemic [24]. Numerous influenza contributions to the WHO’s Global Influenza Surveillance Network through CDC is another example. These contributions have resulted in numerous examples of viruses isolated by DoD’s surveillance network being used as reference strains and the virus seed strain for seasonally available influenza vaccines [25,26].

This global DoD surveillance network should not and does not operate in a vacuum. A review of the DoD-GEIS influenza programs by IOM in 2007, conducted after the first year that the network received avian influenza/pandemic influenza (AI/PI) supplemental funds, commented: “DoD-GEIS should further strengthen its coordination and collaboration on pandemic influenza … with all U.S. partners … These partners include HHS [U.S. Department of Health and Human Services], CDC,…*”*[*27*]. The rapid communication to CDC of the novel H1N1 strains identified by two GEIS partner laboratories before any other public health laboratory (see Table 3) is evidence of the implementation of this recommendation. Though funded partners clearly understand the need for timely processing of samples and expeditious communication, it must be continually reinforced throughout the global surveillance network. Personnel turnover is high, and communication of these ongoing needs is a priority.

**Table 3:** Top 10 accomplishments of the global network, 2009:

1. Conducted active infectious disease surveillance, capacity building, training or outbreak investigations in approximately 92 countries and 500 locations through a global network of partners.
2. Served as the primary source for global avian influenza detection. Of globally reported H5N1 infections, 71 percent (37 of 52) were identified or confirmed at DoD partner laboratories funded by AFHSC-GEIS, with the vast majority being performed at the NAMRU-3 laboratory in Cairo, Egypt.

3. Detected the first four cases of novel A/H1N1 through two partner laboratories, the Naval Health Research Center and the U.S. Air Force School of Aerospace Medicine. Communicated results to the CDC.

4. Supported the diagnostic confirmation of the first novel A/H1N1 cases in 14 countries (Bhutan, Cambodia, Colombia, Djibouti, Ecuador, Egypt, Kenya, Kuwait, Lao People’s Democratic Republic, Lebanon, Nepal, Peru, Republic of the Seychelles).

5. Centrally consolidated over eight laboratory- and region-specific partner reports into an extremely well-received and informative one-page dynamic document of the “Department of Defense Global Surveillance Summary.”

6. Improved infrastructure at 52 laboratories in 46 countries, including eight military and 44 civilian laboratories, with emphasis on influenza, and leveraged capability for other emerging infectious disease initiatives.

7. Sponsored and/or conducted 123 training exercises with more than 3,130 representatives from 40 countries.

8. Responded to more than 76 outbreaks in 53 countries; 24 outbreaks were at U.S. domestic and foreign installations, 36 were in partnership with foreign civilian entities and 15 with foreign militaries.

9. More than 15 reports of first laboratory confirmation of etiologic disease causes in regions where the disease had not been previously reported, including leptospirosis, yellow fever, Q fever, brucellosis, St. Louis encephalitis, Venezuelan equine encephalitis, various rickettioses and other pathogens.

10. Supported partners tested more than 72,000 respiratory samples, of which more than 17,000 (24 percent) were influenza-positive and more than 10,000 (15 percent) were novel A(H1N1).

AFHSC-GEIS: Armed Forces Health Surveillance Center, Global Emerging Infections Surveillance and Response System; DoD: Department of Defense; NAMRU-3: Naval Medical Research Unit Number 3; CDC: Centers for Disease Control and Prevention

## Accomplishments: fiscal year 2009

In its entirety, this special supplement of BioMed Central outlines many of the extensive accomplishments of the global GEIS partner network in 2009. Tables 3 and 4 outline the “Top 10 accomplishments of the global network,” and the “Top 10 specific localized accomplishments.”

**Table 4:** Top 10 specific localized accomplishments, 2009:

1. Of three influenza reference strains provided to WHO (A/California/7/2009, A/California/4/2009 and A/Texas/5/2009) by NHRC and USAFSAM, the A/California/7/2009 was selected as the seed strain.
2. Two biosafety-level 3 (BSL-3) laboratories were commissioned in 2009 at NHRC in San Diego, Calif., and AFRIMS in Bangkok, Thailand; and two BSL-2 laboratories were commissioned, one at the University of Buea, Cameroon, and one on the campus of the Cameroonian Army installation in Yaoundé, Cameroon, under supervision of the Global Viral Forecasting Initiative.

3. NAMRU-3 partners reported the first definitive evidence of human cutaneous leishmaniasis from *Leishmania major* infections in Ghana.

4. AFRIMS published the first report of clinically significant *Plasmodium falciparum* malaria resistance to the potent artemisinin antimalarial drug class, spurring WHO, Bill & Melinda Gates Foundation and host national malaria control officials to institute aggressive measures to contain and eliminate artemisinin-resistant malaria in Southeast Asia.

5. The first documented cases of Venezuelan equine encephalitis, brucellosis, dengue and Q fever in Ecuador were reported by NMRCD-Lima, and the first laboratory-confirmed cases of leptospirosis in the border areas of Thailand and Myanmar were reported by AFRIMS.

6. AFRIMS provided timely outbreak response services to the Nepali National Public Health laboratory, ultimately characterizing (by pulse-field gel electrophoresis) nearly 6,000 cases of multidrug-resistant typhoid fever originating from a single point source, and uniformly quinolone-resistant.

7. NAMRU-3 worked closely with WHO to conduct novel A/H1N1 laboratory diagnostic training for 73 participants representing 32 different countries in a strategic and timely two-week period in May 2009.

8. NEPMU-2, NAMRU-3, and AFHSC collaboratively supported CENTCOM efforts in establishing in-theatre novel A/H1N1 testing and isolation of servicemembers deployed or deploying to sites around the world.

9. The WRAIR/USAMRU-K Malaria Diagnostics and Control Center of Excellence, established in 2003, having trained more than 600 malaria microscopists, established new malaria diagnostics training capabilities in Nigeria and Tanzania, leading to a visit by the president of Tanzania to WRAIR to establish new collaborations between the U.S. Army and Tanzania.

10. NMRCD, as part of its expansive febrile-disease surveillance network in the Amazon basin, published the first comprehensive study of the etiologies of undifferentiated febrile illness in Ecuador, documenting the first laboratory-confirmed cases of Venezualan equine encephalitis, brucellosis, dengue and Q fever in Ecuador.

WHO: World Health Organization; NHRC: Naval Health Research Center; USAFSAM: U.S. Air Force School of Aerospace Medicine; AFRIMS: Armed Forces Research Institute of Medical Sciences; NAMRU-3: Naval Medical Research Unit Number 3; NMRCD: Naval Medical Research Center Detachment; NEPMU-2: Navy Environmental Preventive Medical Unit Number 2; AFHSC: Armed Forces Health Surveillance Center; CENTCOM: U.S. Central Command; WRAIR: Walter Reed Army Institute of Research; USAMRU-K: U.S. Army Medical Research Unit-Kenya

## Publications and presentations

Another metric for success is the number of publications in peer-reviewed journals and presentations given by network partners. An accurate count is difficult because the independent network partners leverage funding from various sources for their initiatives. Nevertheless, 112 manuscripts associated with projects partly or wholly supported by AFHSC-GEIS were published in 2009; the number of poster sessions and presentations at various public and private conferences was far higher.

Broadly speaking, 33 peer-reviewed publications encompassed febrile and vector-borne infections and other infectious diseases; 25 were in the realm of respiratory infections, including influenza; 19 described emerging infections; 18 were associated with malaria; nine were about gastrointestinal infection; seven described antimicrobial-resistant organisms; and one was related to sexually transmitted infections. Though populations under surveillance were often a mixture of military and civilian, 28 of these publications were directly related to U.S. or foreign military populations.

These numbers attest to the scientific rigor with which partners conduct their work, their ability to leverage funding to create a relatively balanced portfolio covering all five pillars of infectious disease threats of military importance, and their emphasis on military populations.

## The way forward: tools for success

### International Health Regulations (2005)

The WHO International Health Regulations, established in 1969, were originally intended to identify several specific diseases of concern (plague, yellow fever, cholera and smallpox) among travelers entering a given country. The events of the past few decades have made it clear that a new paradigm was needed to minimize the global impact of an emerging pandemic and its toll on human life. To this end, the International Health Regulations (2005), or IHR (2005), were formally adopted by the WHO 58th World Health Assembly on May 23, 2005, and took effect on June 15, 2007 [28]. The focus of these new guidelines changed from specific diseases of concern to any event that could be considered a “public health emergency of international concern.” Assessments of current capabilities in countries throughout the world were completed in 2009, and compliance with minimum standards of detection and reporting is required by 2012. Building local capability and infrastructure for compliance is the clear goal in IHR (2005), and the regulations acknowledge and encourage countries and organizations that are able to assist resource-poor countries in their compliance process.

Considerable coordination and communication with in-country ministries, academic institutions and other in-country government assets is done by AFSHC-GEIS global partners. However, collaboration and capacity building conducted by DoD partners is being re-examined to comply with a broader U.S. government response, the National Strategy for Countering Biological Threats, and the IHR (2005) framework. The White House National Security staff is playing an active role in this U.S. government coordination. By conducting our program in coordination with this whole of US Government, then our capacity building, outbreak assistance and facilitating in-country diagnostic capabilities with host countries will meet the objectives of all by a) reinforcing amiable relationships between host-country government public health assets and DoD partners; b) developing the capability to report “public health emergencies of international concern,” whereby the entire global community and DoD learns, and world preparations to minimize impact can proceed in a unified and transparent manner; and c) improving DoD’s situational awareness through close, transparent, trusting relationships with host countries, even if an actual public health emergency of international concern does not occur.

## Military-to-military cooperation and collaboration

As briefly discussed in the biosurveillance landscape section of this paper, many U.S. government organizations are becoming involved in global biosurveillance. The mission of DoD’s overseas laboratories necessitates continued engagement with in-country public health authorities. However, with rapidly increasing involvement of other U.S. government agencies, a unique niche that U.S. uniformed officers throughout the world can and should expand engagement is with their global uniformed counterparts. In many cases, militaries are the major providers of health care in their countries, with abilities that far exceed their civilian programs. Despite political agendas, remarkable progress in facilitating open lines of communication can occur when two researchers or public health professionals, regardless of cultural or economic background, establish mutual rapport for a mutual interest: optimal health of their uniformed service members.

Although many military-to-military lines of communication and collaboration currently exist (Table 5), another mechanism AFHSC used to facilitate increased activities in 2009 began with an expanded relationship with the International Committee of Military Medicine (ICMM). ICMM was established in 1921 by Belgian and U.S. medical officers (Commander Medical Officer Jules Voncken and Captain William Bainbridge) after World War I “revealed the importance of closer cooperation between armed forces medical services worldwide” [29]. With 104 member countries, ICMM is an unbiased, transparent organization with the goals of maintaining and strengthening the bonds between all medical services of member states, promoting medico-military scientific activities, and developing and participating in humanitarian operations.

**Table 5:** Fiscal year 2009 military-to-military partnerships by AFHSC-GEIS (14 countries)

Country	Focus of Collaboration	Nature of Exchange
Cambodia	Influenza surveillance & EID lab training	Standardization of laboratory procedures (QA/QC)
Cameroon	Influenza surveillance & EID lab capability	Influenza & EID reporting capability
Kenya	Influenza surveillance	Influenza reporting capability
Lao People’s Democratic Republic	Influenza surveillance & EID lab training	Standardization of laboratory procedures (QA/QC)
Malaysia	Influenza surveillance & EID lab training	Subject-matter expert
Nigeria	Influenza surveillance & EID lab capability	Influenza & EID reporting
Pakistan	Influenza surveillance & EID lab capability	Subject-matter expert
Peru	Electronic disease surveillance	EID and influenza laboratory
	Influenza & EID lab capability	capacity & training; disease reporting capability
Poland	Influenza surveillance & EID lab capability	Influenza & EID reporting capability
Singapore	Influenza surveillance, EID lab capability & disease surveillance	Standardization of laboratory procedures (QA/QC)
Tanzania	Influenza surveillance & EID lab capability	Influenza & EID reporting capability
Thailand	Unit-based electronic surveillance	EID and influenza laboratory
	Influenza & EID lab capability	capacity & training; disease reporting capability
Uganda	Influenza surveillance	Influenza reporting capability
Vietnam	Influenza surveillance & EID lab training	Standardization of laboratory procedures (QA/QC)

EID: emerging infectious diseases; QA/QC: quality assurance & quality control

Because of its unbiased membership policy, ICMM is the only military organization with a formal in-force memorandum of agreement with WHO. Through direct engagements or indirect facilitation and empowerment with ICMM, opportunities are being explored to work with foreign militaries, to further facilitate IHR (2005) compliance, and to facilitate force health protection and global public health in concert with WHO. Joint initiatives include co-sponsoring a forum titled “Emerging Infectious Diseases: the Military’s Role under International Health Regulations (2005)” in September 2010 in St. Petersburg, Russia, and movement toward development of a military public health network to coordinate and provide access to training, resources, and expertise in public health practice and epidemiologic techniques for member state use.

## Conclusions

U.S. DoD has a long and impressive history of infectious disease research and product development. The GEIS program was developed at a time of need by DoD-sponsored U.S. and overseas research laboratories. The wisdom of establishing improved global DoD EID surveillance capabilities is reinforced by numerous contributions to global outbreaks, most recently the 2009 H1N1 pandemic. The greatly increased interest by other DoD organizations and the U.S. government as a whole also reinforces this wisdom.

For optimal preparedness, surveillance is an ongoing process, not one that is implemented only in times of public health emergency. Sustaining these programs also avoids negative perceptions by foreign governments of U.S. involvement only with the “surveillance priority *du jour*.” The right mix of empowering surveillance activities with capacity building is important to mitigate perceptions of taking but not giving. With the framework of current U.S. government guidelines, such as the National Strategy for Countering Biological Threats and IHR (2005), the world is closer than ever to truly working together on surveillance and control of infectious diseases without consideration of borders.

## Competing interests

To the best of their knowledge, the authors report no competing interests.
